# Enhancing adolescent health: the role of strength and endurance school-based HIIT interventions in physical fitness and cognitive development

**DOI:** 10.3389/fpsyg.2025.1568129

**Published:** 2025-05-12

**Authors:** José Antonio Pérez-Ramírez, Maria Paula Santos, Jorge Mota, Francisco Tomás González-Fernández, Emilio Villa-González

**Affiliations:** ^1^University of Granada, Granada, Spain; ^2^Faculty of Sports-University of Porto (FADEUP), Porto, Portugal; ^3^Laboratory for Integrative and Translational Research in Population Health (ITR), Research Center in Physical Activity, Health and Leisure (CIAFEL), Porto, Portugal; ^4^Department of Physical Education and Sport, Faculty of Sport of Sciences, University of Granada, Granada, Spain; ^5^Department of Physical Education and Sport, Sport and Health University Research Institute (iMUDS), University of Granada, Granada, Spain

**Keywords:** endurance, strength, fitness, attention, concentration

## Abstract

**Introduction:**

Physical activity (PA) is widely known for its outstanding benefits across several health domains including physical, psychological, social, and cognitive in children and adolescents. However, global trends indicate how low levels of PA and physical fitness among adolescents are increasing, with over 80% failing to meet the World Health Organization (WHO) guidelines of 60 min of moderate-to-vigorous physical activity (MVPA) daily.

**Methods:**

This study aimed to evaluate the effects of an 8-week high-intensity interval training (HIIT) intervention based on strength and endurance trainings protocols during physical education (PE) classes on body composition, physical fitness, and cognitive functions in adolescents aged 13–17 years. A quasi-experimental pre-post design was employed with experimental group (EG) and control group (CG).

**Results:**

Results indicated significant improvements in the EG in cardiorespiratory fitness (VO_2_max), strength (hand grip, horizontal jump), and speed-agility (4 × 10 m test). Cognitive outcomes assessed via the D2 attention test revealed notable enhancements in concentration, a reduction in errors, and improved processing speed in the EG relative to the CG (*p* > 0.05).

**Discussion:**

Future studies should explore the long-term impacts of HIIT on cognitive growth and academic achievement, as well as determine the ideal duration and frequency of sessions to optimize benefits in various educational contexts. The results obtained reinforce the use of HIIT in school environments and are further research on its application.

## 1 Introduction

Physical activity (PA) is recognized for its extensive benefits across various domains of health, including physical, psychological, social, academic, and cognitive in children and adolescents (Chaput et al., 2020; WHO, 2020). In particular, moderate-to-vigorous physical activity (MVPA) has been positively correlated with several health indicators, such as improved cardiovascular health, favorable body composition, and better metabolic function (Poitras et al., 2016). However, despite the well-documented advantages of PA, a concerning trend has emerged: fitness levels among adolescents are alarmingly low and have significantly declined over recent decades, constituting a major global health concern. The current epidemiological data show a concerning global trend: over 80% of adolescents have inadequate levels of physical activity and fall short of the minimum recommendations set by the WHO (2020). This widespread pattern of physical inactivity raises significant concerns for public health because it is linked to an increased risk of cardiovascular disease, obesity, and adverse mental health outcomes (Guthold et al., 2020). These findings highlight the urgent need for evidence-based interventions to address this serious public health issue.

Recent studies have begun to explore the specific effects of strength training on various components of executive function. Empirical evidence suggests that there is a very significant correlation between cognitive processes and strength performance in the population and specifically in the adolescent population (Olvera-Rojas et al., 2023). We can, therefore, establish a robustly founded framework for understanding the mechanisms underlying learning and cognitive processes in general. According to the scientific literature, cognitive function constitutes a broad neuropsychological construct that encompasses multiple interrelated neurophysiological processes that allow the encoding, processing, storage and manipulation of information, whether sensory or kinesthetic (Cooper et al., 2016). From a neuropsychological perspective, cognitive architecture is structured into six fundamental domains: mnemonic processes, attentional mechanisms, executive functioning, perceptual processing, psychomotor skills and psycholinguistic skills, each of which presents specific subcomponents (visual, verbal, spatial and auditory modalities). A high-intensity interval training (HIIT) program is an alternative training methodology characterized by the application of short-duration exercise repetitions performed at near-maximal effort (around 90–95% of HRmax). This training approach consists of 45 s to 2–4 min bursts of high-intensity activity, such as cycling or sprinting, interspersed with designated recovery times. HIIT procedures produce equal or better improvements in subsequent cardiorespiratory fitness measures compared to traditional training methods, according to recent literature (Mattioni Maturana et al., 2021). One of the characteristics of HIIT is the low volume of time it requires to produce physiological responses comparable to those observed after conventionally prolonged aerobic training sessions (Buchheit and Laursen, 2013). For instance, Costigan et al. (2016) observed significant improvements in cognitive flexibility among adolescents participating in a high-intensity interval training (HIIT) program that incorporated body-strength exercises. Similarly, Tottori et al. (2019) reported notable enhancements in working memory after four weeks of strength training and HIIT in children aged 8–12 years. Early evidence suggests that incorporating strength exercises within a HIIT regimen may produce unique cognitive benefits compared to traditional aerobic interventions. In line with this a previous study (González-Gálvez et al., 2024) found that a HIIT cardiovascular protocol integrated into physical education (PE) classes improved both body composition and cardiorespiratory fitness in overweight and obese adolescents, outperforming a different PE intervention that showed only limited improvements in lipid composition.

Within the context of PE classes, optimizing the benefits of PA in the shortest possible timeframe is crucial. To achieve this, it is essential to implement effective and efficient strategies to promote PA (Ruiz-Ariza et al., 2017b). However, there remains ongoing debate regarding the most effective type of activity, methodology, intensity, and duration for inducing the most significant and sustained improvements in physical fitness (Costigan et al., 2016; Ruiz-Ariza et al., 2017b). HIIT has emerged as a promising approach, offering a time-efficient way to maximize the benefits of PA while simultaneously enhancing various components of physical fitness (Batacan et al., 2017; Eddolls et al., 2018). Typically consisting of short bursts of vigorous activity lasting between 45 s and 2 min, performed at intensities greater than 85% of maximum heart rate (HRmax), HIIT is interspersed with brief rest periods (Costigan et al., 2016). This approach not only improves physical fitness but also appears to positively impact cognitive functions. According to the scientific literature, one of the key mechanisms responsible for the beneficial effects of high-intensity exercise on cognitive function is the brain-derived neurotrophic factor (BDNF) and catecholamine concentrations induced by high-intensity exercise (90–95% HRmax) (Winter et al., 2007). What is more, the adolescents who practice regular exercise concentrate higher amounts of neurotrophic factors, such as BDNF. Moreover, these adolescents have superior cognitive function compared to obese adolescents (Leahy et al., 2020). This suggests that neurotrophic factors, such as BDNF, play a fundamental role in the cognitive function of young people. Research indicates that both acute and chronic exercise can lead to improvements in executive functions, with meta-analytic studies demonstrating substantial cognitive benefits associated with regular physical activity (Álvarez-Bueno et al., 2017). HIIT interventions, in particular, have shown varying outcomes across different populations and health conditions and using the cardiovascular or the strength training approaches. For example, it was reported a 19.4% increase in VO_2_max following a HIIT program in individuals with cardiometabolic diseases, compared to a 10.3% improvement with moderate-intensity continuous training (Weston et al., 2016) A growing body of literature (Costigan et al., 2016; Faria et al., 2020; Kao et al., 2017; Martínez-López et al., 2018; Stankovic et al., 2023) provides evidence that HIIT protocols yield cognitive benefits in adolescents. A recent meta-analysis compared systematically various PE interventions and concluded that HIIT protocol was the most effective for reducing body mass index (BMI) and improving VO_2_max and 20-meter sprint performance, while strength training demonstrated superior outcomes for enhancing standing long jump performance (Wu et al., 2023). Based on the current literature, we hypothesize that a combined strength and endurance training program within a school-based HIIT framework in PE classes will lead to improvements in physical fitness (PF) variables, including cardiorespiratory fitness (CRF), handgrip strength, and long jump performance. Additionally, we anticipate improvements in cognitive functions, particularly attention and concentration. Thus, the main aim of this study was to analyze the effects of 8-weeks of strength and endurance training in school-based HIIT intervention on physical fitness and cognitive functions in adolescents.

## 2 Materials and methods

### 2.1 Study design

A quasi-experimental pre-post intervention design was used with two groups: a CG and an EG focused on HIIT protocols. The students in the intervention group were assigned according to the class to which they belonged. Both groups belonged to the same educational center and the assignments to the EG were made equivalently. The CG received regular physical education classes taught by a different teacher at the same center, who was blinded to the details of the intervention.

This study was conducted between October and November 2023, during the school year and within the schedule of PE classes, which lasted 2 h per week. The students were already familiar with the teaching methodologies of the teachers and vice versa. In addition, they were familiar with the training protocols and the subjective perception of exertion scale (Borg Scale or RPE). The RPE scale is a simple and reliable tool that allows individuals to quantify the intensity of physical effort during exercise, estimating their own level of effort on a scale from 0 to 10, depending on how hard they feel they are working. To examine the effects of two 8-week physical exercise interventions, aimed at improving physical fitness and executive functions, compared to conventional PE classes, students in the CG and EG were asked to maintain their usual training routines and practices, as appropriate. Importantly, students in the CG did not perform any specific activities related to improving physical fitness during PE classes.

### 2.2 Participants

The present investigation comprised a total sample of 84 secondary education students who participated voluntarily in the study (*n* = 42 in the Control Group [CG], mean age = 15.19 ± 0.74 years; *n* = 42 in the Experimental Group [EG], mean age = 15.03 ± 0.18 years). the following exclusion criteria were implemented to ensure methodological rigor and participant safety: (i) presence of pre-existing medical conditions or physical impairments that could potentially confound experimental outcomes (including, but not limited to, cardiovascular diseases, severe respiratory conditions, or musculoskeletal disorders), (ii) acute or chronic injuries that would preclude full participation in the intervention protocol, (iii) absence of written informed consent from legal guardians or subsequent withdrawal of consent during the study period, (iv) non-attendance exceeding 20% of Physical Education sessions throughout the intervention period, (v) presence of medical conditions requiring pharmacological treatment that could potentially influence physical performance or physiological responses, and (vi) previous participation in analogous intervention studies within a six-month period prior to the commencement of this investigation.

Before the beginning of the study, all participants completed the feasible and validated YAP questionnaire on healthy lifestyle habits, including PA levels, sedentary behaviors and screen time (Segura-Díaz et al., 2021). The adolescents were informed of both the protocol and the objectives of the study. In addition, we provided informed consent to their families since they are minors. Participants were informed that participation was voluntary and that they could withdraw from the study at any time, with the guarantee that the student’s data would not be used in the event of withdrawal. The study followed the ethical standards established in the 1964 Declaration of Helsinki for research involving human subjects and obtained approval from the Research Ethics Committee of the University of Granada (case number 2496/CEIH/2021).

### 2.3 Procedure

#### 2.3.1 Pre-intervention

Before starting the study, the school’s management team was informed about the possibility, need, and objective of the research and intervention. To do so, informed consent was obtained from the students’ families and legal guardians, indicating that the intervention would take place during PE classes and within regular school hours without any additional risks associated with regular PE classes. As a next step, we proceeded to design the intervention protocols for EG. Furthermore, to ensure uniformity, all PE teachers received specific training (5 h) with information, content, explanation of risks, and feedback strategies to ensure student adherence to the program.

**Figure 1:**
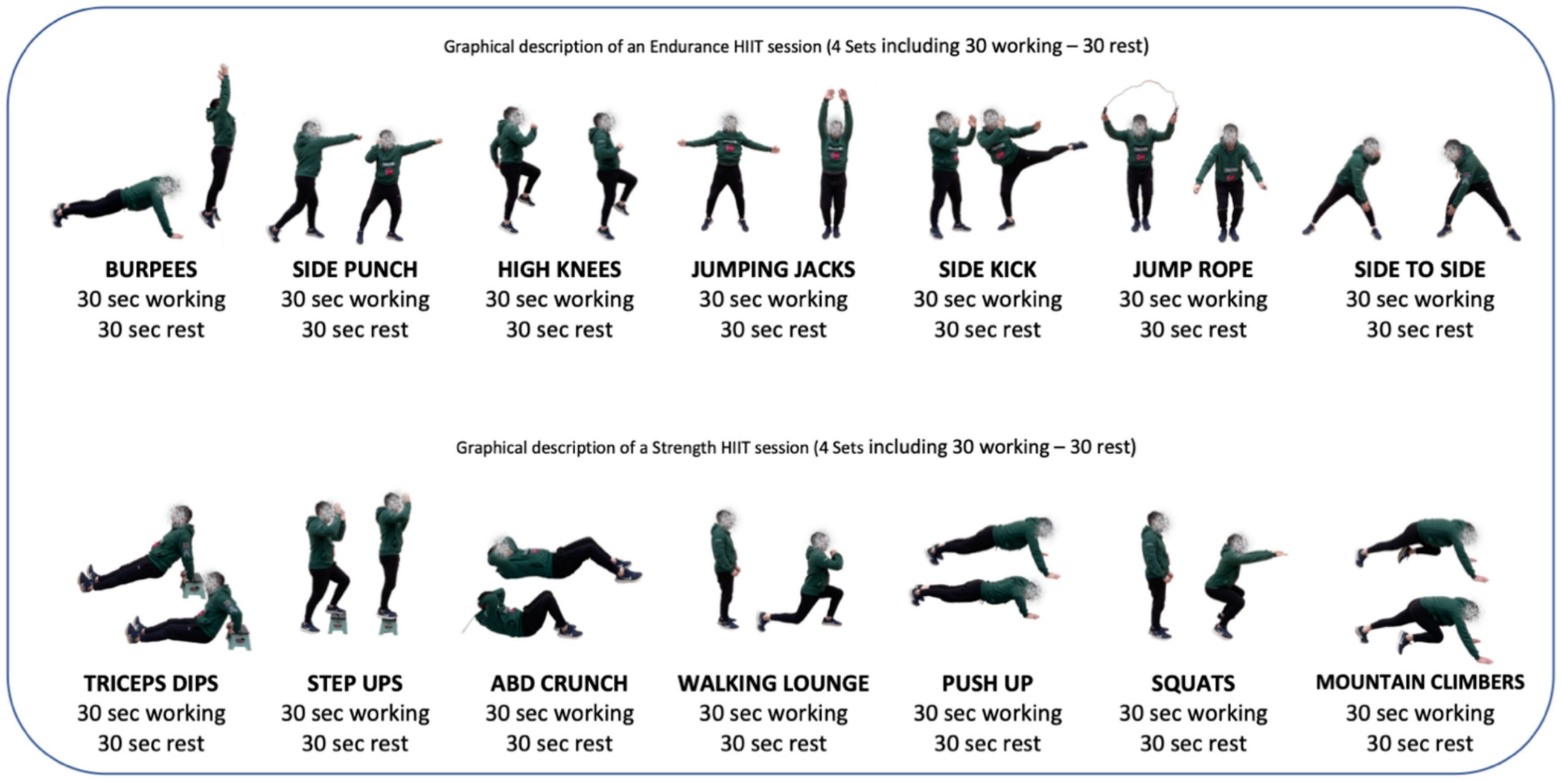
Schematic representation of endurance HIIT and strength HIIT. The sessions consisted of 7–8 exercises with 30 s of high-intensity followed by 30 s resting. The sequences were repeated 4 times each exercise for a total of 35–40 min training session followed by cool-down and retirement of HR monitor.

In sessions prior to the intervention with each group, the D2 test (Brickenkamp, 2002) was presented and explained in detail, resolving the participants’ doubts. Subsequently, anthropometric assessments and a warm-up were performed to facilitate the administration of the ALPHA-Fitness battery (Ruiz et al., 2011) in the following order: long jump without running and speed-agility 4 × 10 meters. In addition, participants were given the necessary recovery time due to the neuromuscular demands in each of the tests used in this session. The sequence of test administration was the same for all participants throughout the study, thus ensuring the standardization of recovery measures. In the second session prior to the intervention, participants performed the Course Navette test after completing the individualized warm-up activities. Measurements were performed during regular school hours. Each task or test was supervised by a researcher, who was also a PE instructor and was properly trained in data collection techniques, especially with regard to the assessment of physical fitness. Before the intervention itself, a series of familiarization sessions with POLAR heart rate monitors and POLAR H10 bands were applied so that each student could see their aerobic and anaerobic threshold individually. Furthermore, during the pretest phase, a series of instructions were given prior to the intervention, as well as during the Course Navette test, to show the students their HR max. In addition, the use of the heart rate monitor during the intervention allowed each subject to moderate their intensity in case they reached frequencies close to the maximum for too long. For more information, please see the Figure 1.

#### 2.3.2 Intervention

The CG students underwent 8 weeks of standard PE classes, whereas the EG engaged in 8 weeks of HIIT focused to endurance and strength interventions within the PE class schedule during school hours. The HIIT protocols focusing on strength and endurance were not conducted in the same session. Instead, they were developed in separate classes, following a standardized routine of two days per week: one HIIT session focused on strength and one on endurance. In total, participants completed 8 strength-based HIIT sessions and 8 endurance-based HIIT sessions. Familiarization with functional training, endurance and strength training through recreational activities and games occurred during initial physical education classes in the first month of the academic year. The training program was designed specifically for physical education teachers and tailored to adolescents. A pilot test confirmed its feasibility and positive student response. The program included engaging exercises, such as martial arts-inspired punch and kicks for cardiovascular improvement and unconventional strength exercises like lifting kettlebells, TRX strap training, Tic-tac-toe game during a plank exercise and heavy wheels to motivate students. Sessions were structured to ensure logical progression in intensity, variety and participant grouping. The participant’s heart rate (HR) was monitored throughout the different sessions to ensure that the appropriate intensity was maintained. Both the CG and the EG were provided with a POLAR Ignite 2 HR monitor and a HR monitor chest strap Polar H10. During the sessions, the HR monitors were given to the students and it was ensured that the students connected the HR monitor correctly and logged in. Afterwards, the general and specific warm-up for each session began. To avoid alterations in the results, the HR monitor data corresponding to the warm-up have been excluded, which corresponds to the first 10 min of each session, both CG and EG. Each PE session in GC and CG lasts 55 min, although it used 40 min of time, since the previous explanations, distribution and logging of the HR monitors, warm-up and cool-down take a certain amount of time, as well as a POLAR IGNITE 2 watch to monitor their HR at all times in order to try to perform physical activity at the highest intensity possible. HR zones were determined using the standard formula HRmax = 220 – age HRmax = 220 − age. Although this method is common in physical intervention studies, we recognize that the use of individualized values (e.g., maximal exercise tests) would have allowed for greater precision in training intensity. For the CG, PE classes predominantly featured cooperation–opposition games and sports emphasizing factors of lesser significance in terms of strength and endurance. It should be noted that in PE sessions time is dedicated to prior explanations, organization of materials and cool-down and stretch phase. It must add that the H10 polar bands of each of the students should be put and removed before and after each PE session. Even so, a maximum of 35–40 min of engagement motor time (EMT) have been reached in some sessions, working with HIIT methodology in all session to maximize EMT. For more information, please, see the Figure 1. During the phase cooling down or stretch part, the pulsometers were removed and a common set was made, also asking about the subjective perception of each student’s effort (RPE), registering this data in a table of each student. Furthermore, music was used in the interventions to increase the motivation and movement of the adolescents, encouraging higher levels of commitment in each PE class and ergogenic values that positively influence physical performance during the task (Aburto-Corona and Aragón-Vargas, 2017; Álamo-Martínez et al., 2024), even better post-exercise recovery (Karageorghis et al., 2018).

#### 2.3.3 Post-intervention

After the eight-week intervention period, both the CG and the EG underwent evaluation within the same week, albeit on separate days, consistent with the two post-interventions, within the same conditions.

### 2.4 Measures

All the following measurements were evaluated pre- and post-intervention.

#### 2.4.1 Anthropometry

For anthropometric measurements, height and weight were measured for each student as part of the anthropometric measurements. To ensure consistent results, the principal investigator used a scale accurate to 0.1 kg to record the weight of each student. Height was measured with a stadiometer (Model SECA 225, Hamburg, Germany) that offered an accuracy of 0.1 cm. Participants were asked to remove their shoes and any accessories that might affect the accuracy of the measurement. For the measurement, they were asked to stand upright, with their arms extended at their sides, looking straight ahead and not moving. Two height and weight measurements were taken for each student, and the average of both measurements was used. All anthropometric measurements, including waist circumference, were taken by the same experienced investigator to ensure consistency in the data. Although no formal indicators of reliability were assessed, the investigator’s vast experience in taking these measurements minimized the possibility of variations.

#### 2.4.2 Physical fitness

The ALPHA-Fitness battery of tests was used to assess the fitness level of the students. In addition, the protocols established for this battery were followed, as well as the guidelines of the American College of Sports Medicine (ACSM) to ensure the safety of the participants. Before the assessment using the battery, all students were informed about the protocols necessary for accurate data collection. Fitness measurements included hand grip strength, which assesses upper extremity strength of the body, and the long jump without running, which assesses lower extremity strength and explosive power. In addition, cardiorespiratory capacity and speed-agility were assessed.

##### 2.4.2.1 Standing long jump

This test assessed the explosive strength of the lower extremities. Each participant had to perform 2 horizontal jumps to reach the maximum possible distance (in centimeters). Each participant performed the long jump without running twice, with a 20-s recovery interval between attempts to mitigate the effects of fatigue. The best jump was recorded as the final result.

##### 2.4.2.2 Speed-agility test 4 × 10 m

This test assessed coordination, agility and speed. Participants were required to complete four repetitions of a 10 m distance, running at top speed. Each participant had two attempts and the best performance was recorded in seconds using a Casio hand-held stopwatch (HS-3 V-1).

##### 2.4.2.3 Course Navette

CRF was assessed using the shuttle run test (Navette Course). Students were required to run between two lines 20 m apart while synchronizing their pace to audio signals broadcast by a loudspeaker following the test protocol. The initial speed was set at 8.5 km/h and increased by 0.5 km/h every minute. The last completed half-day was recorded as an indicator of CRF. In addition, VO2 max was estimated using the equation VO2 max = 5.857 × speed (km/h) – 19.45.

#### 2.4.3 Cognitive functions D2 test

The D2 test (Brickenkamp, 2002) evaluates participants’ capacity to respond to specific visual stimuli. The instrument consists of 14 sequential rows, each containing 47 elements comprising the letters ‘d’ and ‘p’. Participants were allocated a 20-s interval per row to identify and mark instances of the letter ‘d’ that featured two dashes positioned either above or below the character, while disregarding other configurations. Participants were instructed to maintain optimal speed within the designated timeframe, responding to verbal signals indicating row transitions. Performance accuracy was critically important, as both commission and omission errors resulted in score penalties. The D2 test yielded three fundamental metrics: concentration performance, error rate, and processing velocity. The evaluation protocol encompassed multiple parameters: total elements processed (TR), accurate responses (TA), omission errors (O), and commission errors (C). Further analytical components included task efficiency indices (TOT), concentration measures (CON), and comparative analyses between maximum and minimum stimulus processing rates (TR+ and TR− respectively). The protocol also incorporated a variation index (VAR) to assess performance fluctuations between processed stimuli. The instrument demonstrated robust psychometric properties, with test–retest reliability coefficients reaching 0.90 in the original validation study. The concentration metric was computed by subtracting both omission and commission errors from the total processed elements, thus providing a comprehensive measure that integrated both speed and accuracy components. The error parameter quantified incorrectly processed items, while processing speed was determined by the aggregate number of detected elements.

### 2.5 Statistical analysis

Data were analyzed using Statistica software (version 13.1; Statsoft, Inc., Tulsa, OK, USA) and the significance level was set at *p* < 0.05. Normal distribution and homogeneity tests (Kolmogorov–Smirnov and Levene’s, respectively) were conducted on all metrics. Paired sample *t*-test was used for determining differences as a repeated measures analysis (pre–post). Effect size is indicated by Cohen *d* for *t*-test and partial eta squared for Fs. To discover between-group differences, an ANCOVA test was performed using the pretest as a covariate and the times pre and post as factors. In this regard, to interpret the magnitude of the effect size, we adopted the following criteria: *d* = 0.20, small; *d* = 0.50, medium; and *d* = 0.80, large. To interpret the magnitude of the effect size of ANCOVA we adopted the following criteria: *ηp^2^* = 0.02, small; *ηp^2^* = 0.06, medium; and *ηp^2^* = 0.14, large.

## 3 Results

Descriptive statistics were calculated for each anthropometrical measures were evaluated (weight, waist circumference and body mass index), physical fitness (horizontal jump, handgrip, 4×10 m, and V0_2_ max), and attentional variables (TR, TA, O, C, TOT, CON, TR+, and TR-) in each moment of intervention (see Table 1).

**Table 1:** Anthropometrical, performance, and D2 variables before (pretest) and after (posttest) the intervention period (mean ± SD).

Control group (*n* = 42)				Experimental group (*n* = 42)		Differences between groups	
	Pretest	Posttest	RM *t*-test (*p*)	Pretest	Posttest	RM *t*-test (*p*)	Groups (ANCOVA)
Anthropometric measures							
Weight (kg)	59.10 ± 13.47	60.05 ± 13.21	*p* = 0.01*, *d* = −0.07	54.27 ± 13.60	55.80 ± 13.35	*p* = 0.001**, *d* = −0.07	*p* = 0.001, $\eta_{p}^{2}$ =0.98
WC (cm)	69.90 ± 9.70	71.46 ± 9.72	*p* = 0.06, *d* = −0.03	66.43 ± 11.27	71.74 ± 11.21	*p* = 0.001**, *d* = −0.10	*p* = 0.001, $\eta_{p}^{2}$ =0.70
BMI (%)	22.72 ± 4.49	23.30 ± 5.01	*p* = 0.31, *d* = −0.04	21.31 ± 3.93	22.76 ± 8.22	*p* = 0.23, *d* = −0.10	*p* = 0.001, $\eta_{p}^{2}$ =0.24
Physical fitness							
HJ (cm)	131.70 ± 41.42	138.64 ± 33.81	*p* = 0.09, *d* = −0.15	145.14 ± 24.58	151.94 ± 27.45	*p* = 0.001**, *d* = −0.07	*p* = 0.001, $\eta_{p}^{2}$ =0.64
H (kg)	23.01 ± 6.24	23.16 ± 5.79	*p* = 0.66, *d* = −0.02	23.11 ± 6.02	23.86 ± 6.35	*p* = 0.03*, *d* = −0.06	*p* = 0.001, $\eta_{p}^{2}$ =0.88
4×10 (sec)	14.36 ± 2.46	14.75 ± 2.11	*p* = 0.31, *d* = −0.03	14.13 ± 1.69	12.95 ± 1.21	*p* = 0.001**, *d* = −0.11	*p* = 0.001, $\eta_{p}^{2}$ =0.23
CN (V02_max_)	37.16 ± 4.40	37.24 ± 5.38	*p* = 0.70, *d* = 0.00	39.14 ± 5.01	41.12 ± 5.77	*p* = 0.001**, *d* = −0.06	*p* = 0.001, $\eta_{p}^{2}$ =0.63
D2 Test							
TR	348.57 ± 82.37	411.24 ± 66.96	*p* = 0.12, *d* = −0.12	373.81 ± 78.91	430.50 ± 78.48	*p* = 0.001**, *d* = −0.27	*p* = 0.74, $\eta_{p}^{2}$ =0.00
TA	136.661 ± 41.79	152.40 ± 44.45	*p* = 0.01*, *d* = −0.34	132.60 ± 33.33	159.24 ± 30.81	*p* = 0.001**, *d* = −0.42	*p* = 0.001, $\eta_{p}^{2}$ =0.43
O	28.55 ± 34.77	30.86 ± 36.03	*p* = 0.74, *d* = −1.09	26.43 ± 29.69	12.33 ± 12.25	*p* = 0.001**, *d* = 9.97	*p* = 0.03, $\eta_{p}^{2}$ =0.06
C	10.64 ± 21.85	12.00 ± 25.80	*p* = 0.69, *d* = 0.00	7.14 ± 11.33	0.88 ± 2.27	*p* = 0.001**, *d* = 0.00	*p* = 0.001, $\eta_{p}^{2}$ =0.28
TOT	345.38 ± 78.01	387.64 ± 77.57	*p* = 0.001**, *d* = −0.21	339.05 ± 76.10	398.02 ± 68.58	*p* = 0.001**, *d* = −0.35	*p* = 0.001, $\eta_{p}^{2}$ =0.48
CON	125.98 ± 38.20	140.40 ± 46.93	*p* = 0.01*, *d* = −0.40	124.26 ± 37.25	158.36 ± 31.82	*p* = 0.001*, *d* = −0.68	*p* = 0.001, $\eta_{p}^{2}$ =0.50
TR+	35.98 ± 7.00	40.57 ± 6.26	*p* = 0.01*, *d* = −0.18	36.12 ± 7.48	37.74 ± 5.56	*p* = 0.001**, *d* = −0.11	*p* = 0.001, $\eta_{p}^{2}$ =0.18
TR-	18.12 ± 6.34	18.88 ± 8.41	*p* = 0.54, *d* = −0.15	17.55 ± 6.52	20.90 ± 6.38	*p* = 0.001**, *d* = −0.79	*p* = 0.001, $\eta_{p}^{2}$ =0.28
VAR	17.62 ± 6.43	18.98 ± 7.31	*p* = 0.19, *d* = −0.16	18.98 ± 7.31	16.83 ± 6.71	*p* = 0.029, *d* = 0.23	*p* = 0.001, $\eta_{p}^{2}$ =0.15

WC, Waist circumference; BMI, Body Mass Index; HJ, Horizontal Jump; H, Handgrip; 40 ×10, 4 × 10 meters; CN, Course Navette; TR, Processed elements; TA, number of correct responses; O, Omissions; C, Commissions or errors; TOT, Task efficiency; CON, concentration; TR+, stimuli analyzed in the row with the most attempted elements; TR-, fewest attempted elements, and VAR, variation index between the last analyzed stimulus and the differences between them. *Denotes significance at *p* < 0.05 and ** denotes significance at *p* < 0.01.

### 3.1 Heart rate

A t-test with the data from the HR showed higher values in the EG (169.53 ± 2.93) than in the CG (144.54 ± 30.08), *t*(32) = 15,75*, p* < 0.001, *d* = 4.06.

### 3.2 Anthropometric measures

**Figure 2:**
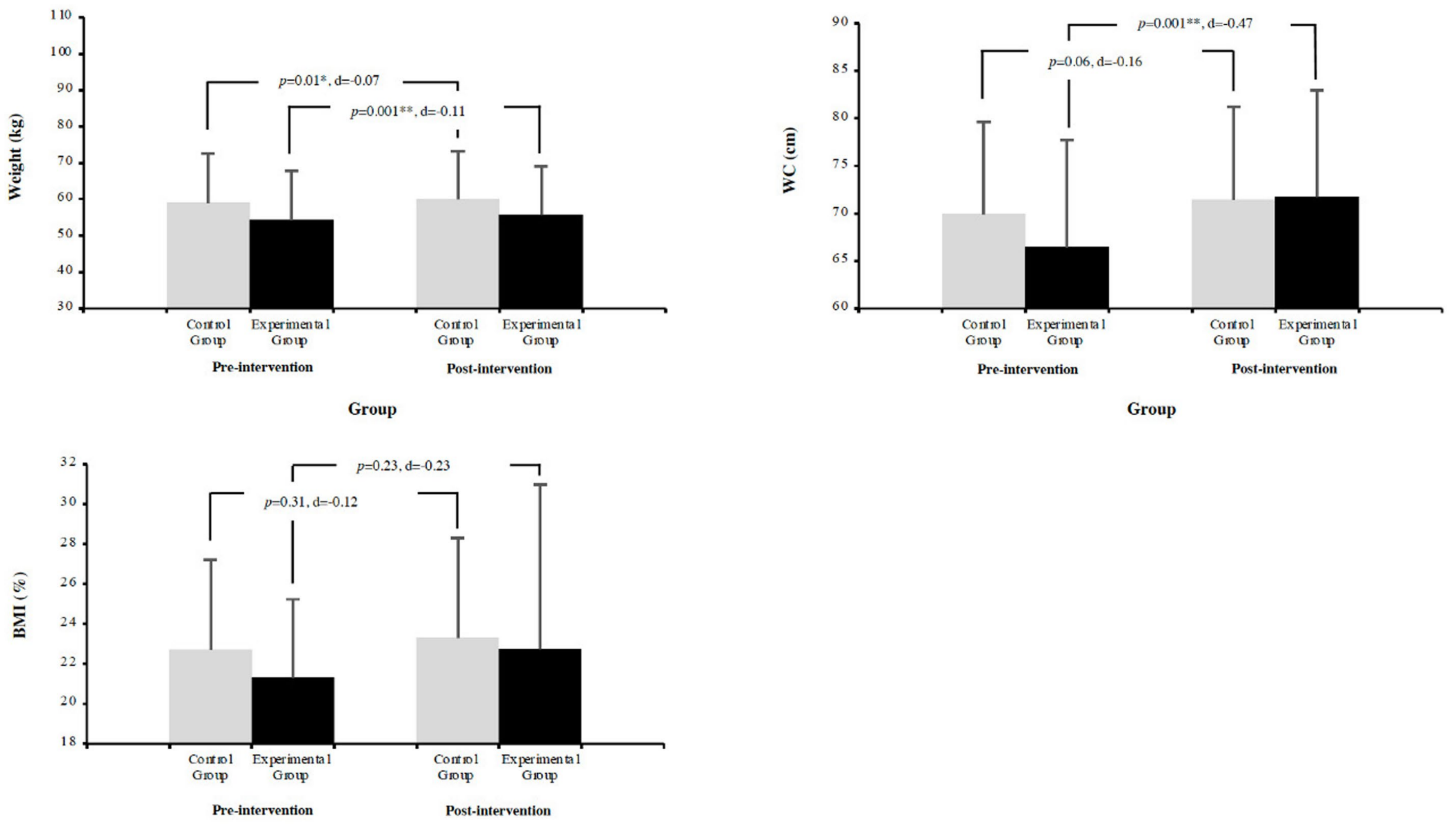
Comparison of pre- and post-test anthropometric measurements between EG and CG.

Repeated measures ANCOVA revealed significant influence of baseline level on Weight, Waist Circumference (WC) and Body Mass Index (BMI) (*p* = 0.001; ηp^2^ = 0.98, ηp^2^ = 0.70, and ηp^2^ = 0.24, respectively). The within-group analysis revealed that EG and GC both groups increased, for Weight, EG significantly increased (2.82%; *p* = 0.001; *d* = −0.07), while CG also significantly increased (1.61%; *p* = 0.01; *d* = −0.07). Regarding WC, EG significantly increased (7.99%; *p* = 0.001; *d* = −0.10), while CG showed a non-significant increase (2.23%; *p* = 0.06; *d* = −0.03). For BMI, EG showed a non-significant increase (6.80%; *p* = 0.23; *d* = −0.10), similar to CG (2.55%; *p* = 0.31; *d* = −0.04). For more information, please see Figure 2.

### 3.3 Physical fitness measures

**Figure 3:**
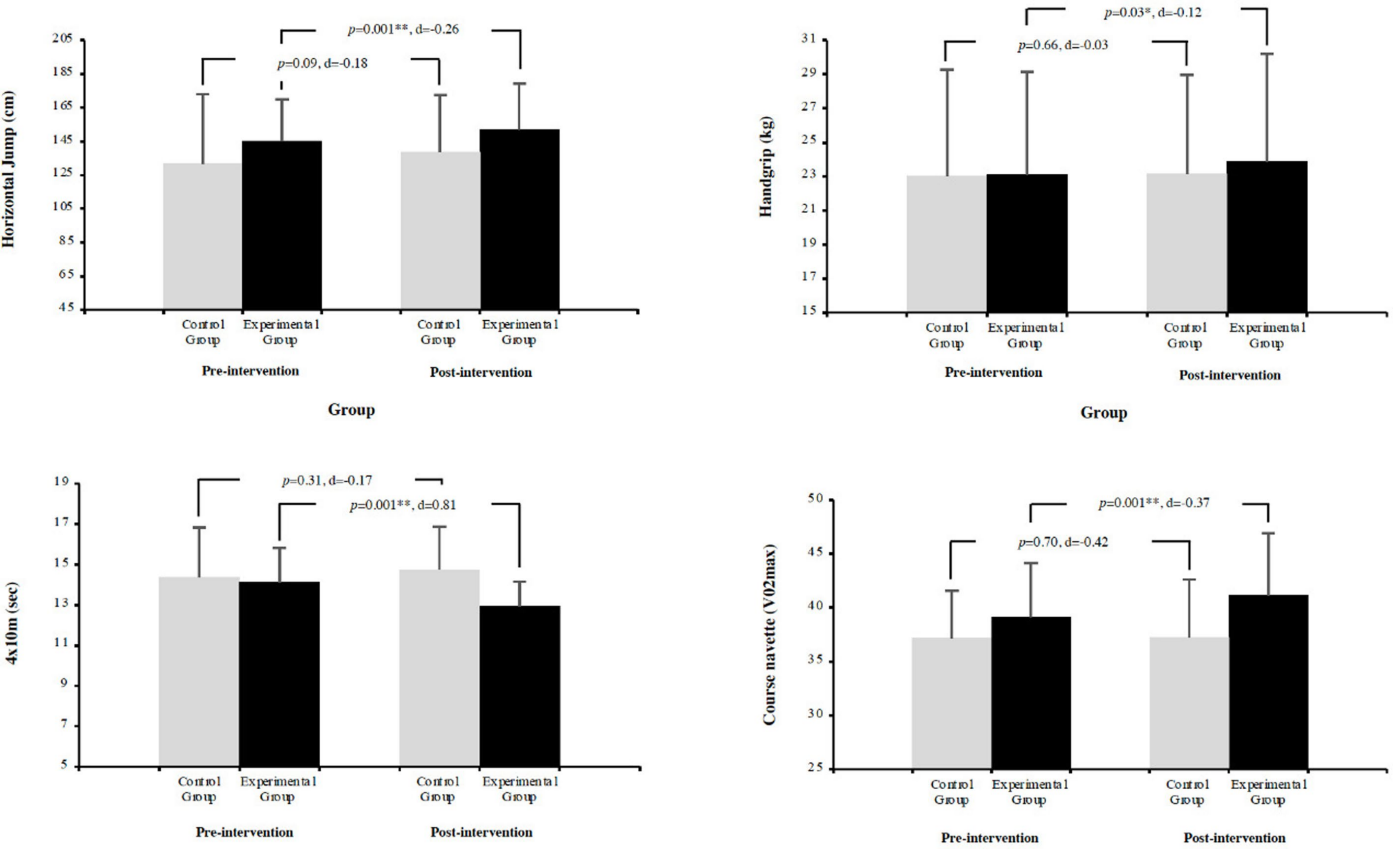
Comparison of pre- and post-test physical fitness measurements between EG and CG.

Repeated measures ANCOVA revealed significant influence of baseline levels on all physical fitness variables (*p* = 0.001; Horizontal Jump: ηp^2^ = 0.64, Handgrip: ηp^2^ = 0.88, 4 x 10m: ηp^2^ = 0.23, VO_2_max: ηp^2^ = 0.63). For Horizontal Jump, EG significantly increased (4.68%; *p* = 0.001; *d* = −0.07), while CG showed a non-significant increase (5.27%; *p* = 0.09; *d* = −0.15). In Handgrip strength, EG significantly improved (3.24%; *p* = 0.03; *d* = −0.06), while CG showed no significant changes (0.65%; *p* = 0.66; *d* = −0.02). For 4x10m test, EG significantly improved by decreasing time (−8.35%; *p* = 0.001; *d* = −0.11), while CG showed no significant changes (2.71%; *p* = 0.31; *d* = −0.03). Regarding VO_2_max, EG showed significant improvements (5.06%; *p* = 0.001; *d* = −0.06), while CG remained stable (0.22%; *p* = 0.70; *d* = 0.00). For more information, please see Figure 3.

### 3.4 Measures of cognition

**Figure 4:**
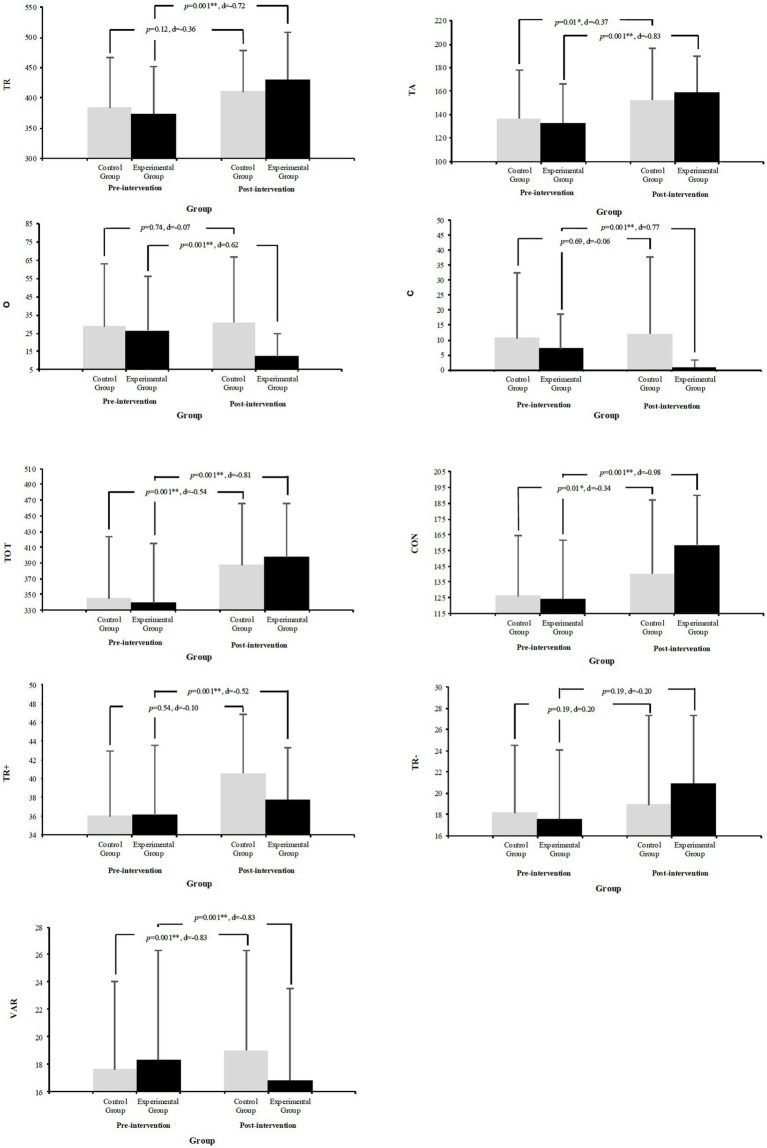
Comparison of pre- and post-test cognitive functions D2 Test between EG and CG.

Repeated measures ANCOVA analysis showed varying effects on attention variables. For Total Responses (TR), no significant between-group differences were found (*p* = 0.74; ηp^2^ = 0.00), although EG showed significant improvement (15.16%; *p* = 0.001; *d* = −0.27) compared to CG’s non-significant change (17.98%; *p* = 0.12; *d* = −0.12). Total Hits (TA) showed significant between-group differences (*p* = 0.001; ηp^2^ = 0.43), with both EG and CG showing significant improvements (20.09%; *p* = 0.001; *d* = −0.42 and 11.52%; *p* = 0.01; *d* = −0.34, respectively). Regarding errors, Omissions (O) showed significant between-group differences (*p* = 0.03; ηp^2^ = 0.06), with EG showing significant reduction (−53.35%; *p* = 0.001; *d* = 9.97), while CG showed non-significant increase (8.09%; *p* = 0.74; *d* = −1.09). Commissions (C) also showed significant between-group differences (*p* = 0.001; ηp^2^ = 0.28), with EG showing significant reduction (−87.67%; *p* = 0.001; *d* = 0.00), while CG showed non-significant increase (12.78%; *p* = 0.69; *d* = 0.007). Total Performance (TOT) showed significant between-group differences (*p* = 0.001; ηp^2^ = 0.48), with both groups showing significant improvements (EG: 17.39%; *p* = 0.001; *d* = −0.35; CG: 12.24%; *p* = 0.001; *d* = −0.21). Concentration (CON) also showed significant between-group differences (*p* = 0.001; ηp^2^ = 0.50), with both groups improving significantly (EG: 27.44%; *p* = 0.001; *d* = −0.68; CG: 11.45%; *p* = 0.01; *d* = −0.40). Finally, TR+ showed significant between-group differences (*p* = 0.001; ηp^2^ = 0.18), with both groups improving significantly (EG: 4.49%; *p* = 0.001; *d* = −0.11; CG: 12.76%; *p* = 0.01; *d* = −0.18). TR- showed significant between-group differences (*p* = 0.001; ηp^2^ = 0.28), with EG showing significant increase (19.09%; *p* = 0.001; *d* = −0.79), while CG showed no significant changes (4.19%; *p* = 0.54; *d* = −0.15). Variation (VAR) showed significant between-group differences (*p* = 0.001; ηp^2^ = 0.15), with EG showing significant decrease (−11.33%; *p* = 0.029; *d* = 0.23), while CG showed non-significant increase (7.72%; *p* = 0.19; d = −0.16). For more information, please see Figure 4.

## 4 Discussion

The present study aimed to evaluate the effects of a strength and endurance training in school-based HIIT intervention, designed to improve physical fitness and cognitive functions in adolescents. The outcomes provide critical information on how a school-based high-intensity exercise intervention can influence these key health parameters. Furthermore, as indicated in the scientific literature, proximately 46% of female and 33% of male adolescents are classified as having inadequate fitness levels, respectively, (Tomkinson et al., 2018). Moreover, more than 80% of adolescents aged 11 to 17 fail to meet the World Health Organization’s (WHO) recommended guidelines for PA, which advocate for at least 60 min of MVPA daily (WHO, 2020).

Results on HR responses revealed differences between EG and CG. As expected, students participating in EG consistently achieved higher HR values (mean = 169.53 ± 2.93) compared to their peers participating in CG (mean = 144.54 ± 30.08), yielding a substantial mean difference of 24.99 points between groups. These findings align with the literature (Duncombe et al., 2023), it observed that young participants routinely reached 79% of their maximum HR, with occasional peaks reaching 92% during similar HIIT interventions. The remarkable consistency of these elevated HR responses across studies strengthens the understanding of how adolescents respond to high-intensity protocols, demonstrated by both a large effect size (η^2^ = 0.82) and impressive reliability coefficients (ω^2^ = 0.81). Furthermore, the marked difference in response variability between groups is striking. The EG exhibited remarkably consistent HR responses, with narrow confidence intervals (167.32–171.73), while the CG showed considerably larger variation (142.25–146.83). This pattern of consistency in HIIT responses is reliable with those obtained by Wu et al. (2023) who noted that well-structured HIIT protocols tend to generate more predictable cardiovascular responses compared to traditional physical activities. These findings build on the comprehensive work of Batacan et al. (2017) whose systematic review highlighted similar patterns of cardiovascular responses in school-based HIIT interventions in this population.

The present study provides valuable insights into the effects of an 8-week HIIT intervention on anthropometric measures of adolescent students, although it is critical to note that improvements in anthropometric measures were not the primary focus of this investigation. The findings reveal interesting patterns that open new perspectives in the field of school-based interventions. The repeated-measures ANCOVA demonstrated a significant influence of baseline values on weight, waist circumference (WC), and body mass index (BMI) (*p* = 0.001), with notable effect sizes (ηp^2^ = 0.98, ηp^2^ = 0.70, and ηp^2^ = 0.24, respectively), providing valuable insights into the role of baseline characteristics in adolescents’ adaptations to exercise. Anthropometric measurements revealed positive increases in both groups that warrant careful consideration within the broader context of adolescent development and uncontrolled variables. The EG showed increases in weight (2.82%; *p* = 0.001; *d* = −0.07), WC (7.99%; *p* = 0.001; *d* = −0.10) and BMI (6.80%; *p* = 0.23; *d* = −0.10), while the CG also showed increases in weight (1.61%; *p* = 0.01; *d* = −0.07), WC (2.23%; *p* = 0.06; *d* = −0.03) and BMI (2.55%; *p* = 0.31; *d* = −0.04).

The research brings to light interesting directions that complement the existing literature. While Hay et al. (2016) observed reductions in body composition after 6 months of intervention, our 8-week program reveals increases in BMI and WC. The patterns observed in this study are in line with the research of Racil et al. (2016) who reported negative changes, i.e., increases after 12 weeks of HIIT intervention and underline the complexity of interpreting anthropometric changes in adolescents. These observations highlight the importance of implementing more comprehensive monitoring systems in school-based interventions that can account for the multiple factors influencing adolescent physical development. These increases could be attributed to several factors inherent to the school intervention context: natural development of muscle mass, normal adolescent growth patterns, which would reveal a positive effect of the increase in BMI and WC, and several uncontrolled external factors such as eating habits, sleeping patterns and extracurricular activities, which would translate into a negative evolution in these variables. The limitation of assessing anthropometric changes without addressing critical variables underscores the necessity for more comprehensive evaluation methods. Recent findings published by Sun et al. (2024) indicate that BMI did not significantly change. Additionally, Olvera-Rojas et al. (2023) highlight that BMI can act as a confounding variable when measuring anthropometric outcomes following strength training interventions.

Several investigations corroborate the findings on the efficacy of physical activity interventions to improve physical fitness. Cardiorespiratory fitness (CRF) is widely recognized as a key indicator of health among adolescents (Tomkinson et al., 2017). Specifically, it was demonstrated significant improvements in inhibitory control among children and adolescents following aerobic exercise interventions (Hillman et al., 2009). Furthermore, acute bouts of PA have been linked to improvements in attentional performance in children (Hillman et al., 2011) highlighting the role of PA in enhancing cognitive abilities, particularly in younger populations. These results showed that baseline levels significantly influenced all physical fitness variables (*p* = 0.001), with particularly strong effects on handgrip strength (ηp^2^ = 0.88) and horizontal jump (ηp^2^ = 0.64). For horizontal jump performance, the EG showed significant improvements (4.68%; *p* = 0.001; *d* = −0.07), while the CG’s increase was not significant (5.27%; *p* = 0.09; *d* = −0.15). This is in line with previous scientific literature that observed expected strength gains of 30–40% in untrained youth after participation in an introductory resistance training program in 8 to 20 weeks (Faigenbaum et al., 2009). Is highlight in the scientific literature, i.e., subjects with a better level of physical fitness are those who experience greater gains because they have greater room for improvement (Guijarro-Romero et al., 2019).

Strength has been shown to yield beneficial adaptations not only in the cardiovascular system but also in cognitive function (Altermann and Gröpel, 2023). Regarding handgrip strength, the EG significantly improved (3.24%; *p* = 0.03; *d* = −0.06), while the CG showed no significant changes (0.65%; *p* = 0.66; *d* = −0.02). These findings align with García-Hermoso et al. (2018) who reported significant improvements in muscle strength after a structured physical activity program.

In the 4x10m test, measuring agility, the EG significantly improved by decreasing time (−8.35%; *p* = 0.001; *d* = −0.11), while the CG showed no significant changes (2.71%; *p* = 0.31; *d* = −0.03). Therefore, in line with the literature reviewed in this study, results similar to those reported by Mitić et al. (2024) were obtained. In their study, a 12-week school-based HIIT intervention led to significant improvements in agility, as assessed by the 4x10m test, as well as an increase in upper body power in the EG compared to the CG, also measured using the 4x10m test agility test. Moreover, in recent years, HIIT interventions in school-based training have shown significant effects on the PF of adolescents (Duncombe et al., 2023). The ALPHA-Fitness battery (Ruiz et al., 2011) validates the importance of this measure for assessing PF in youth.

For cardiorespiratory fitness (VO_2_max), the EG showed significant improvements (5.06%; *p* = 0.001; *d* = −0.06), while the CG remained stable (0.22%; *p* = 0.70; *d* = 0.00). These results are consistent with previous research, as highlighted in a review by Delgado-Floody et al. (2019), which included multiple studies demonstrating significant improvements in cardiorespiratory fitness through various measures including VO_2_max and the Yo-Yo test (Hay et al., 2016; Lambrick et al., 2016; Lau et al., 2015; McNarry et al., 2015; Racil et al., 2016). Similarly, Vieira et al. (2019) found that a 12-week resistance training program significantly improved VO_2_max and aerobic capacity in adolescents. Furthermore Tottori et al. (2019) reported improvements in Course Navette (*p* = 0.042), this study showed a significant increase in VO_2_max (5.06%; *p* = 0.001; *d* = −0.06). It is noteworthy that both studies implemented HIIT protocols of similar duration (8–10 min) and comparable frequency (3 sessions/week), reinforcing the efficacy of this training modality in improving both cognitive and physical aspects in children. Furthermore, the convenience of schools, teaching schedules, and established curriculum made it difficult to incorporate a longer duration of the intervention. Therefore, further research is needed to understand the effects on body composition and physical fitness related to this type of exercise intervention in the school context. Regarding cardiorespiratory capacity, the findings are in the same line with the general trend obtained in the scientific literature.

Analysis of cognitive variables revealed significant effects in reducing omissions and commissions in the sample subjects, as well as improvements in reaction time and a decrease in slow reactions. Specifically, EG demonstrated a greater reduction in omissions and commissions, as well as a decrease in slow reactions. The analysis revealed significant group main effects on cognitive variables. In particular, we observed a significant main effect of time for TR+, indicating temporal improvements in specific cognitive functions. These findings align with the growing body of literature emphasizing the cognitive advantages of PA (Altermann and Gröpel, 2023). Furthermore, a meta-analysis (Sibley and Etnier, 2003) demonstrated the positive impact of physical activity on cognitive function, particularly executive function, in children and adolescents, as demonstrated by another recent study (Rodríguez-García et al., 2022). The outcomes are in line with the effects of physical exercise from other interventions, specifically the effect of cooperative high-intensity interval training (C-HIIT) (Martínez-López et al., 2018). The assessment of cognitive function used measures of attention, specifically focusing on the D2 test (Brickenkamp, 2002) variables: TR, TA, O, C, TOT, CON, TR+, and TR−.

When comparing the outcomes with those reported by Tottori et al. (2019), that found significant improvements in working memory (DSB test, *p* = 0.003) after a 4-week HIIT intervention (3 sessions/week, 8–10 min/session, ≥85% HRmax), this study reveals broader adaptations in attention parameters. Specifically, the EG showed substantial improvements in concentration (27.44%; *p* = 0.001; ηp^2^ = 0.50) and notable reductions in both omission (−53.35%; *p* = 0.001) and commission errors (−87.67%; *p* = 0.001). The results obtained comparing HR of both EG and CG show that the application of a HIIT program in a PE school context has a positive impact on EG cognitive variables in comparison with CG. It may be considered that the volume and intensity of various forms of PA are positively associated with a range of health benefits that extend beyond physical well-being (Chaput et al., 2020). These benefits include enhanced social interactions (Ruiz-Ariza et al., 2017a), improved academic performance (Diamond and Lee, 2011) and better cognitive function (Ruiz-Ariza et al., 2017b). Regarding the HR, EG has an average of 169.53 HR in the sessions and the CG has an average of 144.54 HR. These results indicate a greater intensity of work in EG than in CG whose results coincide with improvements in cognitive variables. These results are in line with recent investigations that have highlighted that high-intensity exercise can stimulate the cognitive functions (Martínez-López et al., 2018; Moreau et al., 2017). Even more, methodologies such as HIIT have emerged, which produce significant improvements in cardiorespiratory fitness and other areas of physical fitness (Eddolls et al., 2018). Furthermore, a systematic review (Wu et al., 2023) comparing various PE interventions and their findings indicate that HIIT is the most effective intervention in reducing BMI and improving VO_2_max and sprint performance, which is in line with the findings.

The practical application of school-based HIIT can be carried out in a fun and effective way through various activities adapted to the level of the students. According to the systematic review by Eddolls et al. (2018), school HIIT programs are much more motivating when they incorporate playful and game elements in these programs, demonstrating significant improvements in the measurement of cardiorespiratory fitness of students participating in these HIIT programs. Researchers Liu et al. (2024) add that HIIT programs that include games and modified sports activities such as small-sized games or altered rules of the game itself to motivate or help students to continue, are effective in improving students’ physical condition. Furthermore, (Lubans et al., 2016) indicated that incorporating intense but short exercises in time such as short sprints, relay games and modified games in HIIT format are much more attractive for students, while promoting health benefits. In addition, the use of HIIT programs can be added to active breaks or even outings to the environment to encourage the use of free time by students, since it has been shown that higher PA in free time is directly related to less use of electronic screen devices (Maher et al., 2019).

The practical implications of the present intervention extend beyond its physical, anthropometric or cognitive outcomes, offering substantial benefits for PE teachers and education professionals. The justification of the study is firmly based on its potential to improve curricular implementation and pedagogical practices in secondary education settings. The findings of this research demonstrate that school-based HIIT protocols can be successfully integrated into the PE curriculum at a legislative level, specifically within the fitness block, without requiring a series of substantial modifications to the established academic planning. This integration represents a practical and major advantage for PE teachers, as it provides scientific evidence-based methodology in line with the current curriculum.

The replication of the intervention offers valuable information for PE teachers in several key aspects. This is curricular integration: the research demonstrates how HIIT protocols can be effectively incorporated into regular PE sessions without altering the established curricular structure or learning objectives and without needing to extend weekly teaching hours during the intervention, i.e., without disrupting the normal functioning of the school or the schedule of the rest of the teaching staff. One aspect to highlight has been to prove to be viable within standard school facilities, including contingency plans for adverse weather conditions, which highlights its adaptability to various educational contexts and resource constraints. Adaptable to any school, student body and context. In addition, the HIIT methodology offers PE teachers an efficient approach to develop students’ physical conditioning within the limited time frame of regular PE sessions. On the other hand, part of our findings indicates that the implementation of the HIIT intervention does not require administrative adjustments of any kind. From a practical perspective, this research provides PE educators with: A structured, evidence-based protocol for implementing HIIT in school, guidelines for managing logistical challenges including shared facility use and weather-related contingencies, and incorporating HIIT without compromising other curricular components. This practical application directly addresses the growing need for effective and time-efficient fitness strategies in educational settings while maintaining alignment with educational standards and institutional constraints, potentially serving as a model for similar interventions in other educational contexts.

The present study has some limitations that provide an opportunity for improvement and further research. First, the research was conducted in a school context, therefore there are uncontrollable variables that may affect the results, such as variability in the implementation of the program such as class schedule and differences in student participation such as motivation, nutrition, sleep hours, etc. The length of the intervention in time (8 weeks, 16 sessions) was conditioned by the school calendar. Furthermore, we can consider the use of more cognitive function measurement tools, even measuring the acute effect of the post-exercise intervention to compare acute and chronic effects in a similar intervention to check the duration of the physiological effect derived from performing high-intensity PA. The implications of this study extend beyond physical education, including areas such as psychology, neuroscience and public health. It promotes interdisciplinary cooperation to investigate the diverse benefits of physical activity, fostering a comprehensive understanding of its influence on students’ overall well-being. In this regard, a strength of this research is its analysis of both physical and cognitive abilities, providing a more comprehensive view of the benefits of physical activity interventions. These findings may motivate policies in the implementation of effective CF improvement programs that address the minimum PA needs recommended by international organizations (Chaput et al., 2020; WHO, 2020). The implications and strengths of the study are that the findings indicate that an exercise intervention with a HIIT protocol can produce varied results in terms of physical and cognitive benefits.

## 5 Conclusion

In conclusion, the implementation of HIIT-based programs in the school context represents an efficient and effective strategy to address current challenges related to low levels of physical activity and fitness in adolescents. Future research could focus on extending the duration of interventions, exploring both the acute and chronic effects of exercise, and considering contextual and motivational factors to optimize outcomes and encourage adherence to physical activity both inside and outside the school environment. The results of this intervention indicate that a number of significant improvements have occurred in key parameters, with the EG displaying improved cardiorespiratory fitness (VO2max improvement of 5.06%), notable cognitive adaptations including improved concentration (27.44%) and reduced attention errors (omissions-53.35% and commissions-87.67%). The intervention results are further complemented by the consistent achievement of target heart rates (mean = 169.53 ± 2.93 bpm) in the EG supporting its efficacy as a time-efficient training methodology. Worthy of note is that this research confirms that HIIT protocols can be successfully integrated into the existing PE curriculum without requiring substantial modifications to academic planning or additional resources, making it a practical and sustainable approach for educational settings. Some practical consequences include the promotion of regular physical activity outside of school hours, as highlighted in other studies, which will encourage students to maintain greater participation in their free time.

## Data Availability

The raw data supporting the conclusions of this article will be made available by the authors, without undue reservation.
